# Genomic insights into virulence and antimicrobial resistance of *Klebsiella pneumoniae* ST25

**DOI:** 10.1128/spectrum.04099-25

**Published:** 2026-09-01

**Authors:** Daniela Cejas, Francisco González-Espinosa, Vincenzo Di Pilato, Gian Maria Rossolini, Marco R. Oggioni, Gabriel Gutkind, Marcela Radice

**Affiliations:** 1 Universidad de Buenos Aires, Facultad de Farmacia y Bioquímica, Instituto de Investigaciones en Bacteriología y Virología Molecular (IBaViM), Ciudad Autónoma de Buenos Aires, Argentina; 2 Consejo Nacional de Investigaciones Científicas y Técnicas (CONICET), Ciudad Autónoma de Buenos Aires, Argentina; 3 Department of Surgical Sciences and Integrated Diagnostics (DISC), University of Genoa 9302, Genoa, Italy; 4 Microbiology Unit, IRCCS Azienda Ospedaliera Metropolitana, Genoa, Italy; 5 Department of Experimental and Clinical Medicine, University of Florence 415681, Florence, Italy; 6 Clinical Microbiology and Virology Unit, Florence Careggi University Hospital, Florence, Italy; 7 Department of Pharmacy and Biotechnology, University of Bologna 201781, Bologna, Italy; 8 IRCCS, Azienda Ospedaliero-Universitaria di Bologna 4488, Bologna, Italy; Instituto de Higiene, Montevideo, Canelones, Uruguay

**Keywords:** *Klebsiella pneumoniae*, ST25, carbapenemase, virulence, resistance, Argentina

## Abstract

Here, we described the resistome and virulome of worldwide *K. pneumoniae* ST25 (Kp-ST25) genomes and established their phylogenetic relationship. Out of a collection of 32 KPC-2-producing Kp-ST25 recovered in Argentina during 2015–2017, eight isolates were selected for whole-genome sequencing (WGS). Additionally, 256 Kp-ST25 genomes were downloaded from NCBI. Bioinformatic analysis was carried out using Unicycler, PROKKA, and Quast. Resistome and virulome were inspected using Kleborate, AMRFinderPlus, BACMET, and VFDB. Phylogenetic estimation included a core genome SNP-based analysis carried out using Roary and IQ-TREE. The tree was visualized using Microrreact. The capsular type K2 was detected in 98% of Kp-ST25 genomes. LPS O1*ab* was the most prevalent, followed by O2*afg* and O3/O3*a*. Most isolates carried the yersiniabactin coding gene, while aerobactin, salmochelin, and RmpADC coding genes were less common. Resistome includes multiple genes for aminoglycosides and quinolones resistance, while 23% of Kp-ST25 harbored carbapenemase-encoding genes (*bla*_KPC-2,-3_; *bla*_NDM-1,-4,-7_; *bla*_OXA-48,-181,-232_). In 37% of Kp-ST25, ESBL-encoding genes (*bla*_CTX-M-2,-15_, *bla*_SHV-12_) were detected as the only acquired *bla* marker. Phylogeny revealed two clades. Clade A is smaller and more virulent, and Clade B is larger and more diverse in virulence and resistance features. Argentinian isolates fall within clade B, showing high resistance and moderate virulence, except one isolate, which displays higher virulence. Furthermore, the Kp-ST25 lineage exhibited intermediate virulence and resistance levels in the genomic analysis.

**IMPORTANCE:**

Hypervirulent *K. pneumoniae* (hv*Kp*) strains cause invasive infections in both healthy and immunocompromised individuals, displaying a more virulent phenotype than classical strains. Apart from lipopolysaccharides (LPSs) and fimbriae, hv*Kp* can present a hypermucoid phenotype due to K1, K2, or K5 polysaccharide capsule and acquired virulence factors encoding the siderophores yersiniabactin (Ybt), aerobactin (Iuc), and salmochelin (Iro), as well as the genotoxin colibactin (Clb). Furthermore, hv*Kp* is becoming increasingly resistant to antibiotics, rendering infection by these strains very challenging to treat. Most of the hv*Kp* isolates belong to sequence types ST23, ST25, ST65, and ST86. Among these, *K. pneumoniae* ST25 was first detected in Argentina in a surveillance study during 2015–2017. These isolates displayed resistance to carbapenems, one of the last-resort antibiotics for Gram-negative complicated hospital infections. The present study contributes to the knowledge of the virulence, resistance, and phylogeny of *K. pneumoniae* ST25 isolated in Argentina, along with others recovered worldwide.

## OBSERVATION

*K. pneumoniae* sequence type (ST) 25 (Kp-ST25) has been described as virulent and hypermucoviscous, often associated with K2 capsular serotype (1–3). The upregulation of capsule production in hypervirulent (hv*Kp*) strains is associated with hypermucoidy loci *rmpADC* and *rmpA/rmpA2* (4, 5). Regarding acquired siderophores, the *ybt* locus is typically located on chromosomal integrative conjugative elements ICE*Kp,* while *iuc* and *iro* loci are encoded in “virulence plasmids.” The presence of *ybt*, *iuc*, and *clb* is used as an overall perspective of the virulence in hv*Kp* (6, 7). Virulence plasmids, carrying *rmpA/rmpA2*, usually also harbor tellurite (*ter*), copper (*pco*), and silver (*sil*) resistance genes, while arsenic (*ars*) and mercury (*mer*) resistance markers have been described in other plasmids (8). In recent years, characterization of hv*Kp* has focused on tracing the emergence of antimicrobial resistance. Carbapenemase-producing Kp-ST25 isolates have been infrequently reported; however, KPC-2, NDM-1, and NDM-7-producing strains have been described as a cause of invasive human infections in Ecuador in 2019, Argentina in 2019, Brazil in 2020, and Chile in 2023 (9–12). Frequently, carbapenem-resistant *K. pneumoniae* isolates co-harbor extended-spectrum β-lactamases (ESBL), such as CTX-M enzymes (13). The presence of coding genes for ESBL and carbapenemases in addition to OmpK mutations, and markers involved in colistin resistance, is used as a resistance level perspective in *K. pneumoniae* (6). This study aimed to describe the resistome and virulome of Kp-ST25 genomes and to establish their phylogenetic relationship.

A total of 264 Kp-ST25 genomes were included in this study. Out of them, eight Argentinian Kp-ST25 isolates (Arg-Kp-ST25) were selected from our collection of 32 KPC-2-producing Kp-ST25 recovered in a previous surveillance study performed in Buenos Aires, Argentina (10). According to the hospital origin of the isolates, strains 6H, 7H, and 14H were randomly selected from Hospital 1, whereas strains 2D, 8D, 17D, 39D, and 44D were randomly selected from Hospital 2. Hypermucoviscosity was evaluated by string tests on 5% sheep blood agar plates, incubated at 37°C overnight in the eight Arg-Kp-ST25 (14).

Genomic DNA obtained by the phenol-chloroform method was subjected to whole-genome sequencing (WGS) on an Illumina MiSeq platform (15). The other 256 Kp-ST25 public genomes were inspected in Pathogenwatch (https://pathogen.watch/) in January 2024 and downloaded from the National Center for Biotechnology Information (NCBI) database.

Unicycler v0.5.0 was used for WGS assembly, PROKKA v1.14.5 for genome annotation, and QUAST 5.2.0 for assembly quality evaluation following Hennart et al. criteria (16). Antimicrobial and metal resistance genes (AMR and HMR, respectively) were predicted using Kleborate v2.2.0, AMRFinderPlus v3.11.26, and Diamond blastX with the BACMET v2.0 database (applying ≥90% of identity and coverage to consider true positives). Virulence factors were predicted using Kleborate and the VFDB database (explored during May 2023). Virulence_scores and resistance_scores were determined using Kleborate (6), which ranged between 0–5 and 0–3, respectively.

cgMLST, cgLIN, and clonal groups were assigned using Pathogenwatch. A core genome SNP-based analysis was carried out using Roary v3.13.0. Phylogeny was inferred using IQ-TREE (GalaxyEU version 2.3.6) under the GTR + F + ASC + G4 evolution model with 1,000 random bootstrap replicates in 264 Kp-ST25 genomes plus one Kp-ST65 genome (a single locus variant of ST25) used as root (Acc. Num. ERR1289816). Microrreact was used to visualize the tree and to present the metadata of the Kp-ST25 (https://microreact.org/).

Associations between the resistance_scores (RS) and virulence_scores (VS) with the main Kp-ST25 clusters identified in this study were analyzed using χ^2^ tests by scipy 1.14.1 and violin plots built by seaborn v0.13.2. Statistical significance was assigned to *P*-values < 0.05.

Arg-Kp-ST25 yielded a negative string test on blood agar. Kp-ST25 genomes (*n* = 264) were mostly from Europe (*n* = 95; 36%) and America (*n* = 78; 30%), with respect to other continents, such as Africa (*n* = 30; 11%), Asia (*n* = 45; 17%), and Oceania (*n* = 16; 6%). K2 capsular type was detected in 259 (98%) of genomes, while the remaining five (2%) genomes were capsule null. Kp-ST25 presented LPS O-type O1*ab* in 249 of them, O2*afg* in 12, and O3/O3*a* in the remaining 3. Chromosomal *ybt* corresponded to *ybt14* located in ICE*Kp5* in 99 genomes, *ybt6*-ICE*Kp5* in 42, *ybt2*-ICE*Kp1* in 34, *ybt16-*ICE*Kp12* in 16, *ybt8*-ICE*Kp9* in 3, *ybt9*-ICE*Kp3* in 2, and *ybt9*-ICE*Kp12* in 1, *ybt4*-plasmid in 2, while 65 genomes were negative for *ybt*. Considering plasmid-borne siderophore systems, *iuc1* was detected in 13 genomes, *iuc3* in 32, and *iuc5* in 2; the remaining 217 genomes did not carry *iuc. iro1* was detected in 13 genomes, *iro5* in 2, a truncated variant of *iro3* in 34, and 215 genomes did not present *iro*. Regarding the *RmpADC* factor, *rmp*1 was detected in 12 genomes, and *rmp3*-ICE*Kp1* was observed in 16 genomes, while 236 genomes did not harbor this factor. The VS_4 (*ybt +iuc*) was assigned in 29 genomes, VS_3 in 18, VS_1 in 170, and VS_0 in 47. Neither isolate fell into the VS_5 nor the VS_2.

Concerning HMR, *ars* was detected in all but one Kp-ST25, *sil* was detected in 229 genomes, *pco* was present in 213, *ter* in 29, and *mer* in 21. Likewise, 212 genomes harbored acquired AMR for aminoglycoside resistance (*aac (3)-IIa*, *aac(6′)-Ib-cr*, *aadA*, *aph3-Ia*, and/or *strB*). *gyrA* mutations (83F, 83Y, 87G, or 87Y) for fluoroquinolone resistance were detected in 32 genomes, and 25 out of them co-harbored the *parC*-80I mutation. Also, 158 genomes presented *qnr*-like alleles. Additionally, 172 genomes harbored *sul,* 176 presented *tet*, and 208 carried *dfrA,* responsible for sulfonamides, tetracycline, and trimethoprim, respectively. Only one genome was positive for *mcr-8*. Sixty-one (23%) Kp-ST25 fell into RS_2, presenting carbapenemase-encoding genes (*bla*_KPC-2-3_, *bla*_NDM-1,-4,-7_, *bla*_OXA-48,-181_, *bla*_VIM-4_, or *bla*_IMP-1_); 97 (37%) Kp-ST25 showed a RS_1 harboring ESBL-encoding genes (*bla*_CTX-M-2,-15_, *bla*_SHV-12_) but non-carbapenemase-encoding genes, while RS_0 was assigned in 106 (40%) Kp-ST25 (absence of ESBL-encoding genes and carbapenemase-encoding genes). Neither isolate fell into RS_3 (carbapenemase-encoding genes + colistin resistance genes).

**Fig 1:**
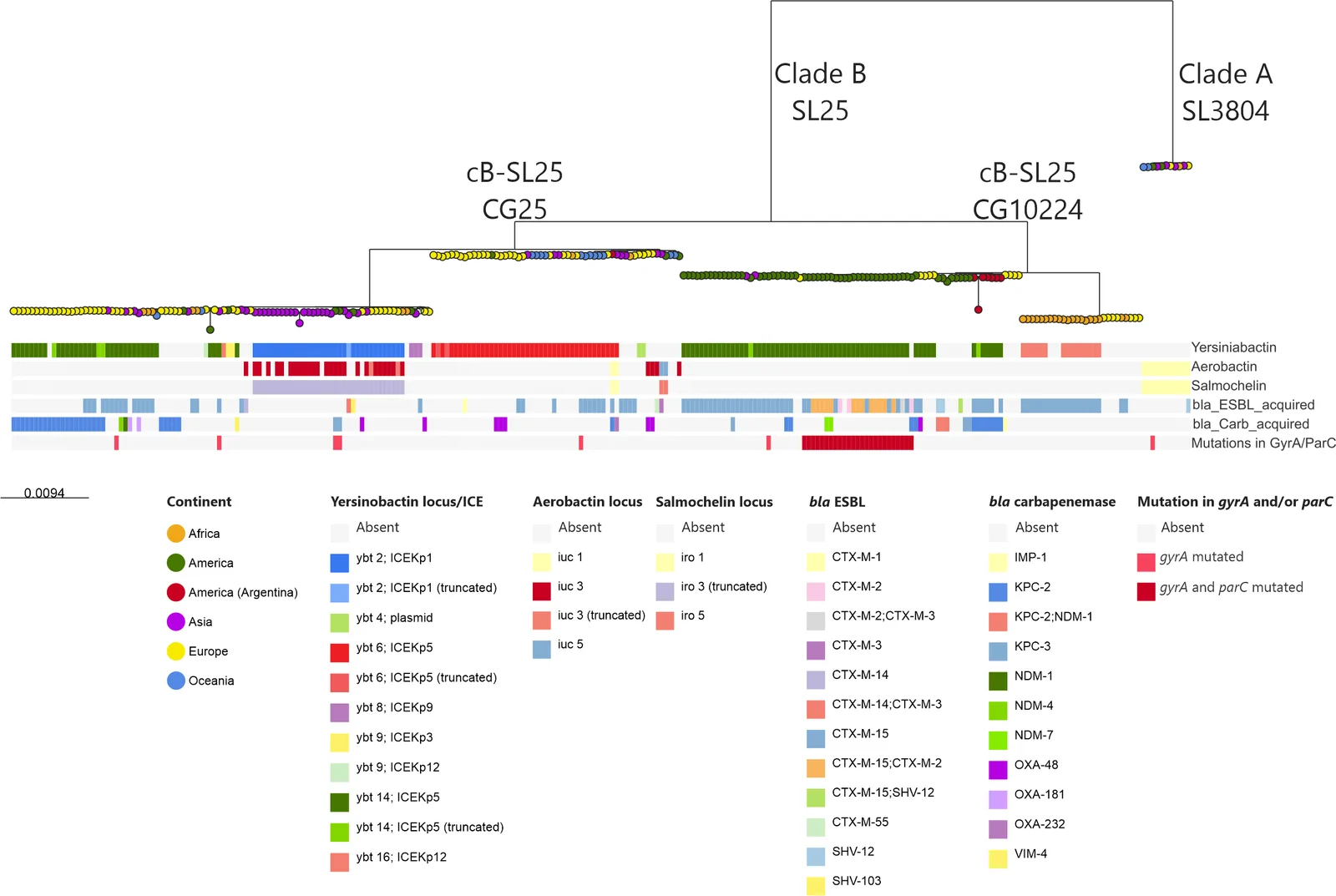
Maximum likelihood phylogenetic tree of 264 *K. pneumoniae* ST25. This tree and the metadata can be viewed in https://microreact.org/project/6VhtepCToNbnWxoJNcW4x3-st25publication.

The phylogenetic tree displayed two main clades (A and B) (Fig. 1). Clade A contained 11 isolates recovered in diverse geographical origins corresponding to the single lineage (SL) and clonal group (CG) 3804 (cA-SL3804). They presented the *repB*_Kleb replicon consistent with the KpVP-1 archetypal virulence plasmid and the hypermucoidy gene *rmpA* (4). Most of them carried *ter*. Non-ESBL-encoding genes nor carbapenemase-encoding genes were detected in this group; however, two isolates carried *bla*_DHA-1_.

Clade B grouped 253 genomes, which corresponded to SL25 containing CG10224 and CG25 (cB-SL25-CG10224 and cB-SL25-CG25, respectively). cB-SL25-CG10224 comprised 103 genomes, divided into two main branches. One of them contained the majority of genomes from the Americas (65/78), including 43 from South America (Argentina, *n* = 7; Colombia, *n* = 11; Chile, *n* = 25). The second branch grouped 27 genomes from Europe and Africa. Meanwhile, cB-SL25-CG25 was divided into two branches. One comprised 56 genomes from diverse geographical origins, but mostly from Europe, and included the remaining Argentinian genome (39D). The second branch included 94 genomes, predominantly from Asia.

**Fig 2:**
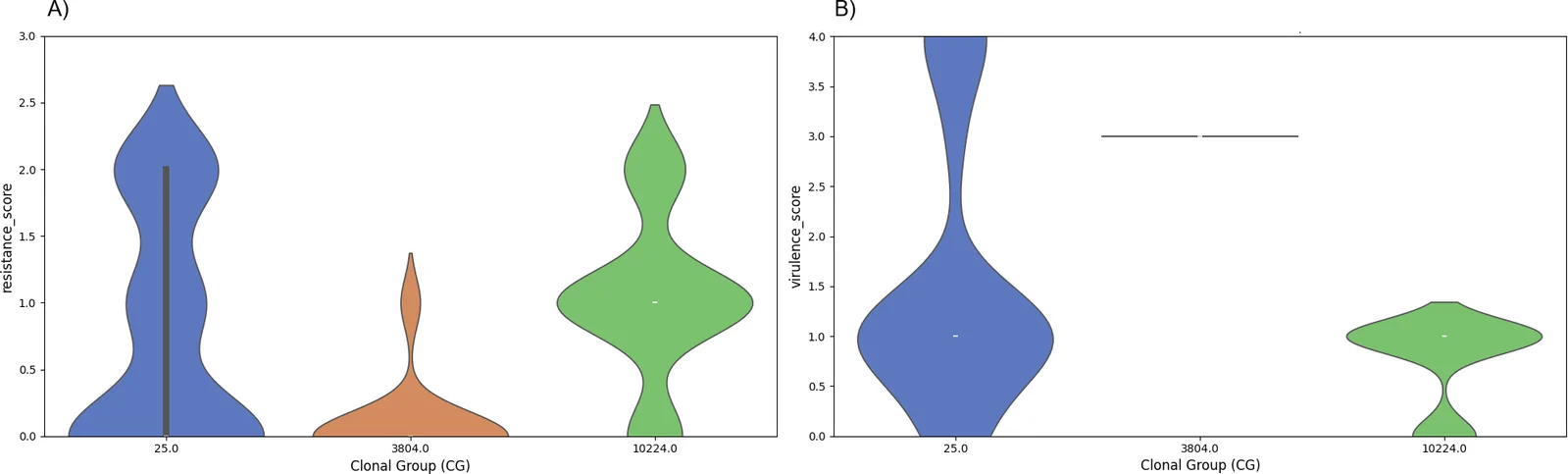
Associations between the resistance, virulence, and the main clusters of *K. pneumoniae* ST25. (**A**) Resistance scores (RS) distribution by clonal groups. (**B**) Virulence scores (VS) distribution by clonal groups.

Figure 2 shows that RS dispersion was similar for cB-SL25-CG25 and cB-SL25-CG10224, ranging among the scores 0-3. The median corresponded to the RS_1 for cB-SL25-CG10224, while the median for cB-SL25-CG25 and cA-SL3804 corresponded to the RS_0. cB-SL25-CG10224 showed a higher RS than cA-SL3804 or cB-SL25-CG25 (χ^2^, *P*-value = 2.1e−12). On the other hand, VS dispersion was different between the three groups, being the largest in cB-SL25-CG25 and the lowest in cA-SL3804, which exhibited all statistical measures in VS_3. The median was similar for cB-SL25-CG10224 and cB-SL25-CG25, falling at VS_1. cA-SL3804 carrying *iuc1* displayed a higher VS than the other two (χ^2^, *P*-value = 6.1e−37).

Slightly more than a third of Kp-ST25 were ESBL producers displaying RS_1. All Arg-Kp-ST25 harbored *bla*_KPC-2_ (10), as well as 30 isolates from Colombia, Chile, and the United Kingdom; most assigned to RS_2 (*bla*_KPC-2_ + ESBL-encoding genes).

The LPS O1 type, reported as the most predominant in clinical isolates, was present in the vast majority of Kp-ST25. The subtype O2*afg*, the second most frequent among Kp-ST25, had been associated with an improved survival in human serum factors (17). The most prevalent variant of siderophore systems was *ybt14* (also present in Arg-Kp-ST25), which was associated with ICE*Kp5* for its mobilization. Most isolates carrying *ybt2-*ICE*Kp1* co-harbored *iuc3*, a needed aerobactin marker for successful infection by hv*Kp* in mouse infection models (18).

On the other hand, *ars* was ubiquitous in Kp-ST25, while *pco* and *sil* were the other two most frequent HMR. The tellurite resistance gene was detected among a few Kp-ST25.

Phylogenetic analysis exhibited three important clusters (cA-SL3804, cB-SL25-CG10224, and cB-SL25-CG25) that presented consistency with cgMLST, cgLIN, and CG (Fig. 1). Isolates from the same geographical area did not cluster together; however, South American genomes located in cB-SL25-CG10224, being the only genome separate from these, the Argentinian 39D, which grouped in cB-SL25-CG25. Genomes from Europe clustered with those from Africa and Asia across different branches, potentially reflecting human mobility between these regions; however, this should be interpreted with caution, as no travel-related epidemiological metadata were available or analyzed in this study (19).

The distribution of Kp-ST25 was similar between both clusters of cB-SL25, while cA-SL3804 contained a few genomes compared with the other two. The cB-SL25-CG10224 (containing most of KPC-2-producing Arg-Kp-ST25) correlated with higher RS, while the more susceptible was cA-SL3804. On the other hand, the content of virulence factors was higher in isolates clustered in cA-SL3804. Kp-ST25 isolates exhibited different virulence and resistance trends; however, the highest virulence or resistance score has not been detected in any cluster. According to Kochan et al., convergent isolates (carrying both resistance and virulence determinants) frequently display lower virulence than pure hv*Kp* in mouse models, despite representing an increased clinical risk (20). Although no *in vivo* studies were performed here, the ST25 lineage exhibited intermediate virulence and resistance levels in the genomic analysis, consistent with a divergence between these traits. In summary, MLST determination alone is insufficient to infer a hypervirulent phenotype.

## Data Availability

Genomes were deposited in the NCBI Bioproject database (PRJNA954772 and PRJNA1202099).
